# Feasibility of self-testing for acute malaria using rapid diagnostic tests in three peri-urban sub-Saharan African community settings

**DOI:** 10.1186/s12936-025-05727-6

**Published:** 2025-12-19

**Authors:** Amanda Brumwell, Meagan Bemer, Amber Lauff, Jennifer F. Morton, Katherine K. Thomas, Caitlin A. Moe, Shawna Cooper, Hilton Humphries, Anjali Sharma, Derek Pollard, Zachary Kwena, Norton Sang, Anna Winters, Alastair van Heerden, Elizabeth Bukusi, Dino Rech, Paul Drain

**Affiliations:** 1 Department of Global Health, University of Washington, Seattle, USA; 2 University of California Irvine, Irvine, USA; 3 Audere, Seattle, USA; 4 Human Sciences Research Council (HSRC), Pretoria, South Africa; 5 Centre for Infectious Disease Research in Zambia, Lusaka, Zambia; 6 Akros, Lusaka, Zambia; 7 Kenya Medical Research Institute (KEMRI), Nairobi, Kenya; 8 Department of Obstetrics and Gynecology, University of Washington, Seattle, USA; 9 Vanderbilt University Medical Centre, Nashville, USA; 10 School of Medicine, University of Washington, Seattle, USA; 11 Department of Epidemiology, University of Washington, Seattle, USA; 12 University of California San Francisco, San Francisco, USA

**Keywords:** Malaria, Rapid diagnostic tests, Self-testing, SSA, Feasibility, Acceptability, Usability

## Abstract

**Background:**

Malaria is a significant cause of under-five child mortality in sub-Saharan Africa (SSA). The World Health Organization (WHO)-approved rapid diagnostic tests (RDT) for malaria offer a resource-efficient alternative to gold-standard diagnostic methods and may improve timely access to care through self-testing.

**Methods:**

The feasibility of use of RDT for self-testing was evaluated in 100 households each in Migori County, Kenya; KwaZulu-Natal Province, South Africa; and Copperbelt Province, Zambia. Surveys assessed perceived usability, acceptability, and preferences for RDTs among consenting participants.

**Results:**

Among 225 participants in Kenya, 80 in South Africa, and 163 in Zambia, 25 (11.5%), 0 (0.0%), and 3 (1.8%) tested positive for malaria, respectively. In Kenya and Zambia, 89% of participants reported previous malaria diagnoses. Participants across all three sites interpreted the RDT with 100% sensitivity and 99.7% specificity compared to RDT interpretation performed by a trained study team member, with only one individual interpreting their test incorrectly. Over 96% of participants across all three sites felt the RDT would be easy to use for specimen collection, test operation, and result interpretation, and 160 (100%) Kenyan participants, 74 (96.1%) South African participants, and 157 (99.4%) Zambian participants felt confident that they had interpreted their own test correctly. Participants’ perceived comfort for future self-testing with an RDT was high in Kenya (92%) and Zambia (86%), and moderate in South Africa (66%).

**Conclusions:**

These findings indicate that RDT self-testing is highly acceptable and feasible in SSA settings with a high malaria burden.

## Background

In 2022, approximately 223 million malaria cases and 580,000 malaria deaths occurred in the World Health Organization (WHO) African Region, accounting for 94% of the global malaria burden [1]. Although the region has seen a 60% decline in malaria mortality and 40% decline in incidence between 2000 and 2022 [1, 2], the burden remains particulary high for children under the age of five, for whom disease progression and death may occur rapidly[3]. Globally, the malaria mortality rate among children under 5 has stagnated since 2015, despite improvements in malaria incidence over the same period[4]. For those who survive, malaria is associated with numerous severe sequelae, such as kidney and spleen damage, anemia, and neurological complications.

Timely identification of malaria is critical for initiating artemisinin-based treatment within 48 h of symptom onset to reduce the risk of disease progression and complications [5]. However, obtaining a diagnosis by blood smear microscopy or polymerase chain reaction (PCR) remains challenging due to lack of technical expertise and specialized laboratory equipment, particularly in rural settings or areas where access to facility-based care is limited [6]. Malaria Rapid Diagnostic Tests (RDTs), whose use has been approved by the WHO for primary diagnosis, are increasingly used to identify malaria in healthcare settings [7]. As of 2019, RDTs were used to identify malaria for 87% of cases in African settings using RDTs [4, 8, 9]. However, due to limited access to RDTs, providers rely on clinical diagnosis of malaria based on non-specific symptoms such as fever, nausea, and muscle pain [1]. Also, the WHO recommends that in malaria-endemic settings, malaria should be suspected in anyone presenting with a fever and antimalarial treatment initiated if diagnosis is not available within two hours [5]. These proactive approaches overlook malaria in individuals who are asymptomatic, have low parasitological counts, or who are unable to present to clinical care [10]. As a result, malaria is paradoxically both over-treated and under-diagnosed in sub-Saharan Africa (SSA) [1, 10, 11], underscoring the need for rapid, reliable, and accessible diagnosis [1].

While RDTs are increasingly used in clinical settings, many people may continue to have poor access to clinical services, which may increase their risk of developing severe malaria [12]. Moreover, resource-limited health systems may struggle to meet the demand for malaria diagnosis and treatment due to lack of sufficient training, personnel, equipment, and anti-malarial medication [13, 14]. Although the WHO has recommended that RDTs be leveraged in community-based settings to broaden access to diagnostic services [5], the feasibility of implementing RDTs for self-testing at the community or household level, remains unclear [6, 15]. Notably, many SSA settings have established networks of community health workers (CHWs) that deliver health services closer to where people live and work, and which may be leveraged to improve community members’ access to timely diagnosis and care. However, less understood is the feasibility of self-testing in the absence of a trained CHW. To address this diagnosis gap and determine the feasibility of future, population-level malaria elimination initiatives including the possibility for self-testing, the community-based usage of RDTs was piloted in three countries in sub-Saharan Africa (SSA).

## Methods

### Study objective and aims

This study’s objective was to evaluate the feasibility of a decentralized testing package, including malaria RDTs, in three SSA community settings. The first aim was to describe the results of RDT testing for malaria at the household and individual levels, considering age, location, and other demographic characteristics. The second aim was to characterize the acceptability, usability, and user preferences for RDTs.

### Selection process

A cross-sectional study of individuals belonging to households was conducted in the following settings: Umsunduzi, KwaZulu-Natal, South Africa; Migori County, Kenya; and Luanshya District, Copperbelt, Zambia. Umsunduzi, South Africa is a predominantly isiZulu-speaking area with no domestic malaria transmission [16]. Migori County, Kenya is home to a population of about 1 million people with high rates of malaria transmission that contribute significantly to under-five child mortality [17, 18]. Copperbelt Province, Zambia is a primarily Icibemba-speaking area with a high burden of malaria transmission [19]. In each of these settings, selected communities contained an estimated population between 200 and 2000 in a high density peri-urban and/or rural area.

One hundred households were selected in each of Kenya, South Africa, and Zambia. In South Africa, a community map was generated and each household or dwelling structure was identified as a visiting point that may be selected for inclusion. Visiting points were randomly sampled, and in cases where more than one household was present at the visiting point, a Kish grid was used to select one household for participation [20]. In Kenya, the county was divided into five diverse community settings: peri-urban areas, fishing communities, gold mining zones, agricultural regions, and among individuals engaged in the informal transportation sector (e.g. motorcycle taxi or “boda boda” drivers). From each of these five zones, CHWs selected 20 households. In Zambia, 100 households were systematically sampled through approaching every third household in the community. Individuals in the selected households were eligible to participate in this study if they were six months of age or older, able to provide consent or assent, and resided in the selected household. Written consent was obtained from all individuals 18 years or older. For participants under 18, age-appropriate assent was obtained, and written consent was obtained from participants’ guardians or parents.

### Intervention

As RDTs had not been approved for self-testing at the time of the study, all participants were offered observational malaria self-testing regardless of symptom status. The Abbott Bioline Malaria Ag P.f RDT and the Abbott Bioline Malaria Ag P.f/Pan RDT were used in Zambia and South Africa respectively, and both RDTs were used in Kenya (Abbott Diagnostics, Chicago, USA) based on the local market availability. Each participant was provided an orientation to the RDT testing components and included step-by-step instructions for the testing process, including how to interpret the RDT results. A study team member trained according to standard operating procedures performed the RDT, as these tests were not approved for self-testing at the time of the study. As the study team member performed the RDT, they narrated the process to the participant. The study team member asked the participant to interpret the RDT results; the study team member would then verify the results with their own interpretation of the RDT, and these results were compared for consistency. Individuals who tested positive for malaria received counselling and referral to a health facility for diagnostic confirmation and follow-up, per local guidelines.

Using a tablet-based REDCap data collection software [21, 22], research assistants administered a survey collecting: (1) age and occupation, (2) history of malaria diagnosis, (3) history of past RDT usage, (4) usability of the RDT, (5) acceptability of RDT, and (6) preferences for RDT usage. Acceptability was measured using a five-point Likert scale as the degree to which the participant would be willing to self-test without the assistsance of a health worker [23]. Usability was measured using three, five-point Likert scale questions assessing the participant’s judgement for the ease of collecting the testing specimen, applying the testing specimen to the RDT, and matching and interpreting results [24].

### Analysis

Using descriptive statistics, age was summarized in medians and interquartile range (IQR), as well as the frequency of individuals testing positive for malaria, stratified by WHO-defined age groups for malaria (under 5 years old, 5–15 years old, and over 15 years old) [5]. Prevalence of previous malaria diagnoses were similarly presented by age groups and study sites.

The number of households with at least one person testing positive for malaria was calculated to evaluate RDT positivity. The potential clustering of cases was examined by finding the number of individuals with a positive malaria test per household. A generalized estimating equation (GEE) logistic regression model, which generates confidence intervals that account for potential correlation in malaria positivity within household, was fit to estimate overall malaria RDT positivity in each country given that participants were sampled by households. Sensitivity and specificity of participant RDT interpretations were calculated compared to those of trained study team members.

Feasibility is defined as the extent to which a new treatment or innovation can be successfully used or carried out within a given agency or setting [23]. For this study, feasibility is a product of the external characteristics of a setting in which an intervention is conducted; the preferences of the intervention recipients, including their perceived acceptability of the intervention; and the recipients’ interaction with the intervention. Leveraging early conceptual frameworks to assess the feasibility of RDTs [25], feasibility was measured as (1) potential RDT self-testers’ perceived usability of the test, defined as the degree to which participants deem the test to be easy to use [24], and (2) acceptability of the test, defined as whether an individual would be comfortable self-testing without the help of a health worker [23]. Perceived usability was assessed by collapsing the Likert scale responses into negative or affirmative categories and analysing them as frequencies and percentages. Reported acceptability and preferences for use of RDTs were assessed using descriptive statistics.

Household level feasibility was evaluated by calculating the frequency and percentage of households with at least one individual indicating (1) overall usability (affirmative responses to all usability questions) and (2) acceptability (an individual’s comfort performing an RDT independently). Analysis was performed using R (version 4.3.3).

### Ethics approval

This study was approved in Kenya by the Kenya Medical Research Institute’s Scientific and Ethics Review Unit (Reference KEMRI/SERU/CMR/P00229-011–2022/4701), and research license granted by the Kenyan National Commission for Science, Technology and Innovation. In South Africa, this study was approved by the Human Sciences Research Council Research Ethics Committee (Reference REC 8/23/11/22). In Zambia, this study was approved by ERES Converge (Reference 2023-May-004).

## Results

Among the 100 households enrolled at each study site (300 total), 596 participants were enrolled: 261 in Kenya, 148 in South Africa, and 187 in Zambia (Table 1). In Kenya, 195 adults and 66 children (defined as < 18 years old) were enrolled in the study; 140 adults and 8 children were enrolled in South Africa, and 176 adults and 11 children were enrolled in Zambia. Over half of participants identified as female: 135 (52%), 97 (66%), and 136 (73%) in Kenya, South Africa, and Zambia, respectively. The median age of adults was 35 (IQR: 24.5–47), 38 (IQR: 28–58), and 43 (IQR: 27.75–60.25), and the median age of children was 7 (IQR: 3.25–10), 15.5 (IQR: 15–16.25), and 13 (IQR: 9–16), in Kenya, South Africa, and Zambia, respectively. In Kenya, the most common occupation after being a student (51, 19.5%) was farming or agriculture (47, 18.0%). In South Africa, most participants were unemployed (106, 71.6%), and 17 (11.5%) were students. In Zambia, 53 (28.3%) individuals were unemployed, 41 (21.9%) were employed in small market sales or trade work, and 35 (18.7%) worked in the farming or agriculture industry. The majority of participants had not previously performed a RDT on themselves or on their child.

**Table 1:** Demographics and previous RDT usage

	Kenya		South Africa		Zambia		Total	
	N	%	N	%	N	%	N	%
Total households	**100**	**–**	**100**	**–**	**100**	**–**	**300**	**–**
Total participants	**261**	**–**	**148**	**–**	**187**	**–**	**596**	**–**
Adults	195	74.7	140	94.6	176	94.1	511	85.7
Children (< 18 years old)	66	25.2	8	5.4	11	5.9	85	14.3
Female gender	135	51.7	97	65.5	136	72.7	368	61.7
Median age in years (IQR)								
Adults	35 (24.5–47)		38 (28–58)		43 (27.75–60.25)		37 (27–54)	
Children	7 (3.25–10)		15.5 (15–16.25)		13 (9–16)		8 (4–12)	
Occupation								
Child or minor but not a student	32	12.3	0	0	3	1.6	35	5.9
Farming/Agriculture	47	18.0	0	0	35	19	82	13.8
Housewife	9	3.4	7	4.7	3	1.6	19	3.2
Manufacturing/Factory	0	0	4	2.7	2	1.1	6	1.0
Office or clerical work	1	0.4	0	0	0	0	1	0.2
Other manual labor	4	1.5	2	1.4	29	15.5	35	5.9
Small-market sales or trade	9	3.4	1	0.7	41	21.9	51	8.6
Student	51	19.5	17	11.5	19	10.2	87	14.6
Unemployed	6	2.3	106	71.6	53	28.3	165	27.7
Other	102	39.1	11	7.4	2	1.1	115	19.3
Participant has ever performed a rapid diagnostic test to self-test for any condition								
Total	196	75.1	146	98.6	182	97.3	524	87.9
Yes	33	16.8	32	21.9	7	3.8	72	13.7
No	163	83.2	113	77.4	175	96.2	451	86.1
Unsure/Don’t know	0	0	1	0.7	0	0	1	0.2
Parent or guardian has ever performed a rapid diagnostic test on their child								
Total	65	24.9	2	1.4	5	2.7	72	12.1
Yes	5	7.7	0	0	0	0	5	6.9
No	60	92.3	2	100	5	100	67	93.1

Demographics and RDT usage in Kenya, South Africa, and Zambia. Total: 300 households (596 participants). Adults: 85.7%, children: 14.3%. Female: 61.7%. Median age: adults 37 years, children 8 years. Occupations: farming (13.8%), unemployed (27.7%), students (14.6%). Self-testing: 13.7% self-tested of those responding had used RDTs previously. Child-testing: 12.1% parents responded regarding ever having performed RDTs on children; 6.9% confirmed yes

In Kenya, 225 (86.2%) participants agreed to a malaria test, of whom 25 (11.1%, 95% CI 7.3–17.0%) tested positive (Table 2). Of the 160 individuals over 15 years old tested for malaria, 16 (10.0%) tested positive. All 41 Kenyan participants between 5 and 15 years old were tested, of whom 5 (12.2%) were positive, and all 24 Kenyan participants under 5 were tested, of whom 4 (16.7%) were positive for malaria. In South Africa, 80 (54.0%) participants agreed to be tested for malaria; this included 76 of the 144 (52.8%) participants over 15 years old, and all 4 of the participants between 5 and 15 years old, none of whom tested positive. Three (1.8%) participants in Zambia tested positive (95% CI: 0.6–5.5%), all of whom were over 15 years old. When an RDT was not performed, the most common reason at all three sites was that the participant declined testing. Among participants who were not tested, 25 (69.4%) participants in Kenya, 65 (95.6%) participants in South Africa, and 22 (91.7%) participants in Zambia chose not to be tested.

**Table 2:** Results of testing for malaria and past experiences using RDTs

			Kenya		South Africa		Zambia	
			n/N	%	n/N	%	n/N	%
Tested for malaria	All participants	Total	**225/261**	**86.2**	**80/148**	**54.1**	**163/187**	**87.2**
		Positive	25	11.1	0	0	3	1.8
		Negative	200	88.9	80	100	160	98.2
	Over 15 years old	Total	160	71.1	76	95.0	158	96.9
		Positive	16	10.0	0	0	3	1.9
		Negative	144	90.0	76	100	155	98.1
	5–15 years old	Total	41	18.2	4	5.0	5	3.1
		Positive	5	12.2	0	0	0	0
		Negative	36	87.8	4	100	5	100
	Under 5 years old	Total	24	10.7	0	0	0	0
		Positive	4	16.7	–	–	–	–
		Negative	20	83.3	–	–	–	–
Previous malaria diagnosis	All participants	Total	**261/261**	**100**	**148/148**	**100**	**187/187**	**100**
		Yes	233	89.3	0	0	166	88.8
		No	24	9.2	148	100	19	10.2
		Unsure	4	1.5	0	0	2	1.1
	Over 15 years old	Total	196	75.1	144	97.3	181	96.8
		Yes	176	89.8	0	0	162	89.5
		No	17	8.7	144	100	17	9.4
		Unsure	3	1.5	0	0	2	1.1
	5–15 years old	Total	41	15.7	4	2.7	6	3.2
		Yes	35	85.4	0	0	4	66.7
		No	5	12.2	4	100.0	2	33.3
		Unsure	1	2.4	0	0	0	0
	Under 5 years old	Total	24	9.2	0	0	0	0
		Yes	22	91.7	–	–	–	–
		No	2	8.3	–	–	–	–
Positive malaria tests per household		0	79/100	79.0	100/100	100	97/100	97.0
		1	19/100	19.0	0/100	0	3/100	3.0
		2	0/100	0	0/100	0	0/100	0
		3	2/100	2.0	0/100	0	0/100	0
Not tested for malaria		Total	36/261	13.8	68/148	45.9	24/187	12.8
		Participant declined	25	69.4	65	95.6	22	91.7
		Could not get enough blood from finger prick	0	0	0	0	1	4.2
		Stockouts	0	0	3	4.4	0	0
		Other	11*	30.6	0	0	1^†^	4.2

*Seven (19.4%) participants who were not tested indicated they were currently on malaria treatment, and four (11.1%) indicated they were tested recently

^†^Participant indicated they were currently on malaria treatment

Malaria testing results and past experiences in Kenya, South Africa, and Zambia. Testing rates: Kenya 86.2%, Zambia 87.2%, South Africa 54.1%. Positivity: Kenya 11.1% (25 cases), Zambia 1.8% (3 cases), South Africa 0%. Children under 5 had highest positivity in Kenya (16.7%). Previous malaria diagnosis: Kenya 89.3%, Zambia 88.8%, South Africa 0%. Households with ≥ 1 positive test: Kenya 21%, Zambia 3%, South Africa 0%. Main reason for not testing: participant refusal (69–96% across sites)

In Kenya, 233 of 261 (89.3%) individuals had been diagnosed with malaria at least once previously (Table 2) including 176 of 196 (89.8%) over 15 years old, 35 of 41 (85.4%) between 5 and 15 years old, and 22 of 24 (91.7%) under 5 years old. In Zambia, 166 of 187 (88.8%) participants had previously been diagnosed with malaria, including 162 of 181 (89.5%) over 15 years old and 4 of 6 (66.7%) between the ages of 5–15. In South Africa, no participants had previously been diagnosed with malaria. In terms of household-level clustering of malaria diagnoses, more than one person tested positive for malaria in three (3%) households in Kenya.

Across all three sites, almost all participants were able to correctly interpret their RDT results. Participants in Kenya had 100% accuracy interpreting their RDT results, with 100% sensitivity and specificity compared to trained study team members (Table 3). South African participants reported their results with 100% specificity, with no estimate for sensitivity since there were no individuals positive for malaria. In Zambia, participant-interpretation of RDT results had 100% sensitivity, due to all three positive tests correctly identified, and 99.4% specificity, due to one participant wrongly interpreting their RDT result as indeterminate rather than negative.

**Table 3:** Sensitivity and specificity of RDTs, comparing participant interpretation to trained professional interpretation as reference

	Kenya	South Africa	Zambia	Total
Sensitivity (participant positives among trained team member positives)	17/17 (100%)	–	3/3 (100%)	20/20 (100%)
Specificity (participant negatives among trained team member negatives)	143/143 (100%)	77/77 (100%)	154/155 (99.4%)*	374/375 (99.7%)

*For one result, the participant interpreted the test as indeterminate rather than negative. No results were considered indeterminate by the trained team member performing the test

Accuracy of participant-interpreted malaria rapid tests compared to professional interpretation. Sensitivity (correct positive identification): 100% in Kenya (17/17), Zambia (3/3), and overall (20/20). Specificity (correct negative identification): 100% in Kenya (143/143) and South Africa (77/77), 99.4% in Zambia (154/155), and 99.7% overall (374/375). *One negative result inccorectly interpreted as an indeterminate result occurred in Zambia

**Fig. 1:**
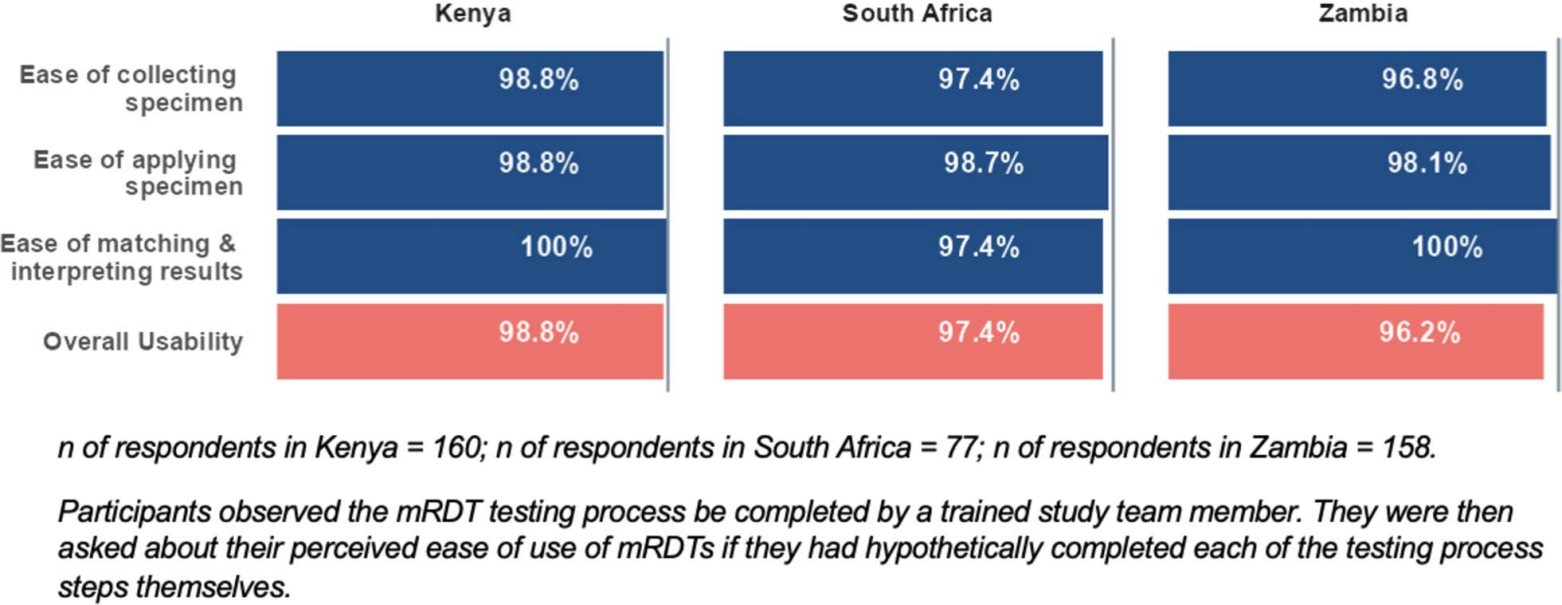
Ease of use of RDTs by country. Three bar charts ease of use data for each of the three sites showing that overall usability of RDTs is above 96%

Of the 160 participants in Kenya who responded to questions about malaria testing, 158 (98.8%) thought it would be easy to collect the specimen (Fig. 1) and to apply the specimen to the testing device, and 100% thought it was easy to match the results in the instructions to those on the device for interpretation. 98.8% of participants indicated overall usability of the RDT.

In South Africa, 75 of 77 (97.4%) participants thought it would be it easy to collect the specimen for the test and to apply the specimen to the testing device. Seventy-five (97.4%) thought it was easy to match the results for interpretation. Seventy-five (97.4%) participants indicated overall usability of the RDT.

In Zambia, 153 of 158 (96.8%) participants who responded to questions about malaria testing thought it would be easy to collect the specimen, 155 (98.1%) thought it would be easy to apply the specimen to the device, and all participants (100%) found it easy to match the results for interpretation. One-hundred-fifty-two (96.2%) participants indicated overall usability of the RDT.

Among participants who answered questions about future testing preferences, 180 of 195 (92.3%) in Kenya, 95 of 143 (66.4%) in South Africa, and 156 of 182 (85.7%) in Zambia indicated that they would be comfortable using an RDT involving a finger prick without the assistance of a health worker (Table 4). The majority—160 of 160 (100%) in Kenya, 74 of 77 (96.1%) in South Africa, and 157 of 158 (99.4%) in Zambia—were confident that they had chosen the correct interpretation for their RDT result. Over 80% of participants at all three sites indicated that they would prefer to use an RDT at home, with secondary preference for conducting an RDT in clinical settings.

**Table 4:** Acceptability of and preferences for RDTs

		Kenya		South Africa		Zambia	
		n/N	%	n/N	%	n/N	%
Confident that the chosen result is the correct one		160/160	100	74/77	96.1	157/158	99.4
Comfortable doing a rapid test involving a finger prick alone without a health worker to help		180/195	92.3	95/143	66.4	156/182	85.7
Where participant would prefer to do an RDT in the future	At home	168/195	86.2	141/143	98.6	150/182	82.4
	In a clinical setting (e.g., at a local health clinic or hospital)	15/195	7.7	1/143	0.7	30/182	16.5
	No preference/Anywhere	12/195	6.2	1/143	0.7	0/182	0
	Unsure/Don’t know	0/195	0	0/143	0	2/182	1.1

Malaria rapid test preferences across three countries. Confidence in results: 96–100%. Comfort with self-testing with a finger prick without a health worker to help: South Africa 66.4%, Zambia 85.7%, Kenya 92.3%. Preferred testing location: Home (82–99%) over clinics (0.7–17%). Kenya had 6.2% with no location preference; Zambia had 1.1% unsure

Among households where at least one malaria test was performed, 91 (98.9%) households in Kenya, 54 (98.2%) households in South Africa, and 86 (97.7%) households in Zambia had at least one participant whose responses to the usability questions indicated overall usability of the RDT (Table 5). As a measure of household-level acceptability, 98 (98%), 70 (71.4%), and 91 (91%) households in Kenya, South Africa, and Zambia, respectively, had at least one participant who indicated that they would feel comfortable performing a rapid test alone without the aid of a health worker.

**Table 5:** Household usability and acceptability of RDTs

	Kenya		South Africa		Zambia	
	n/N	%	n/N	%	n/N	%
At least one participant had a malaria test	94/100	94.0	55/100	55.0	88/100	88.0
At least one individual indicated overall usability of the RDT	91/92*	98.9	54/55	98.2	86/88	97.7
At least one individual comfortable doing a rapid test involving a finger prick on their own, without the help of a health worker	98/100	98.0	70/98^†^	71.4	91/100	91.0

*Among households where at least one person had a malaria test and responded to related questions, two households in Kenya did not have any respondents

^†^Among households where at least one person had a malaria test and responded to related questions; two households in South Africa did not have any respondents

Household malaria test usability in three countries. At least one participant in the household had a malaria test: Kenya 94%, Zambia 88%, South Africa 55%. At least one individual in the household indicated overall usability of RDT: 98% in Kenya, 98% in South Africa, 98% in Zambia. At least one participant was comfortable doing a finger prick rapid test without a health worker’s help: Kenya 98%, Zambia 91%, South Africa 71%. *2 Kenyan households, ^†^2 South African households lacked respondent data

## Discussion

In this community-based study in Kenya, South Africa, and Zambia, use of RDTs was feasible based on the high degree of usability and acceptability. Across all three settings, more than 96% of participants felt that RDTs would be easy to use and had confidence in their interpretation of their test results. Most participants across all three settings also indicated that they would be comfortable using RDTs on their own without a health care provider, which would mirror self-testing conditions. Although there were few positive results in Zambia and none in South Africa which together limit the precision of this study’s estimates, participants were nonetheless able to interpret their RDTs with nearly 100% accuracy. There is, therefore, strong reason to believe that use of RDTs for self-testing would be feasible in these settings.

Of the three sites, participants in Kenya had the highest burden of malaria with 11.1% testing positive, including 16.7% of children under 5. While this is lower than the 34.1% of children under 5 testing positive in rural Kenyan outpatient clinics, heterogeneity across geographical areas and differences in community-based versus facility-based sampling likely drives the difference [26]. Moreover, the all-participant positivity identified in this study is slightly higher than the 9.9% positivity found in a large prospective study in Eastern Kenya [27]. Participants in Zambia had the next highest malaria burden of the three study sites, with 1.8% of participants testing positive. Notably, this is substantially lower than the 19.5% prevalence reported by Zambia’s 2015 national prevalence survey [28]. In both Kenya and Zambia, 89% of participants had been diagnosed with malaria at least once in the past, indicating a high incidence of disease. Given that KwaZulu-Natal has low levels of local transmission of malaria, it was unsurprising to observe no positive RDT results among study participants in this area [16].

In areas with a high burden of disease, early identification of malaria is crucial for quickly initiating treatment, particularly for children under 5 years old, who are at high risk of serious illness and death [5, 29]. Moreover, in high-burden settings, self-testing using RDTs is particularly advantageous for simplifying the cascade of care for patients, reducing the workload on facility-based healthcare workers, and equipping symptomatic individuals with the knowledge to make informed decisions about their health [30, 31]. For RDTs to effectively bolster the capacity of the health system to meet the burden of disease, users must be able to operate RDTs correctly and accurately. Participants could interpret an RDT accurately compared to a trained healthcare worker: all participants correctly identified a positive test result, and all but one participant correctly identified a negative test result, although it is worth noting that sensitivity and specificity were calculated from a very small number of positive tests in the case of Kenya and Zambia. These results echo studies from high-income settings where non-clinicians were found to correctly use RDTs for self-testing [32].

Not only were participants able to interpret RDTs accurately, but more than 95% of participants in all sites also considered these tests to be usable. Nearly all participants felt that collecting a fingerpick sample would be easy, that applying the specimen to the device would be easy, and that it was easy to match the device results to those in the instructions for interpreting the RDT. Ease of use is an important enabler for individual-level willingness to use RDTs, which in turn contributes to the feasibility of broader, population-level uptake [25].

Overall, participants demonstrated a high degree of acceptability towards RDTs, as indicated by their willingness to undergo testing with an RDT and their reported comfort in using an RDT on their own. In Kenya, where the highest prevalence of malaria was observed, a majority (92.3%) of participants indicated that they would be comfortable using an RDT on their own and without the assistance of a health worker. Zambia had a similar level of acceptability (85.7%). South Africa had comparatively fewer people indicating comfort using a RDT (66.4%) or confidence in their interpreted results (96.1%). While these are still indicate overall high rates of acceptability for RDT self-testing, these results were surprising given that more South African participants indicated having used an RDT on themselves in the past. It is possible that these results are reflective of the lower familiarity with malaria in KwaZulu-Natal, rather than with the RDT process itself. Interestingly, few participants had used an RDT on themselves or their children across all study settings; the large proportion of individuals who indicated acceptability in using a RDT may be indicative of participants’ values toward and need for RDTs.

Finally, the majority of participants at all sites indicated that they would prefer to use an RDT at home. This underscores the potential for self-testing with RDTs in these peri-urban and rural, Sub-Saharan African settings that may benefit from decentralized models of care for deadly diseases. It is important to note that while this analysis focused on feasibility of RDTs specifically, it is not sufficient to focus on RDTs in isolation as a solution to malaria. Rather, self-testing with RDTs must be delivered as part of a system that makes care accessible and efficiently links individuals who have a positive test to clinical services. Moreover, should programmes introduce RDT self-testing as a key component of parasemitological identification, it will be important that RDT tests are available and accessible in high-malaria burden communities. Future initiatives might consider testing programmatic designs for RDT provision, including direct delivery to beneficiaries, or whether it is feasible to place the responsibility of procurement and purchasing RDTs on community members. Each delivery design will incure different long-term costs or benefits, including the cost of RDT units as well as the cost of treatment, and which may be borne by programmes, community members, or a combination of the two. Further research is necessary to determine the benefits and costs of different mechanisms to provide RDTs.

Community-based delivery of RDTs may improve access to care, particularly in resource-limited settings [33]. This may have the dual advantage of facilitating patients’ decision-making upon receipt of a positive parasitological confirmation of malaria and improving the efficiency of care by ensuring that patients receiving antimalarial treatment are those who truly have malaria [34]. Examples of community-based RDT applications have relied on the use of RDTs by CHWs or trained staff in pharmacies [15, 35]. This is in contrast to this study, which sought to assess the feasibility of malaria RDTs for self-testing. An important distinction between CHW-administered testing with RDTs and this study’s focus, malaria RDT self-testing, is the means by which individuals can be linked to care in the event of a positive test. CHW-led testing with RDTs improve connectivity to health services, and may facility rapid linkage to care if an individual tests positive in a community setting. In contrast, self-testing may increase access to care by reducing individuals’ reliance on CHW to deliver tests, yet there remain important questions around how individuals may make health-related decisions and access care upon receiving a positive test. Morever, self-testing for other infectious diseases such as HIV [36], influenza [37], sexually transmitted infections [38], and COVID-19 [39] is acceptably accurate compared to gold-standard diagnoses through clinical and/or laboratory-based evaluation. Self-testing for these diseases is also generally highly acceptable [40–43], and malaria’s less stigmatized nature compared to these diseases [44] gives reason to hope that community members may reliably self-test for malaria.

The use of malaria RDTs in community-based settings may bring about other benefits, such as a reduction in the unnecessary prescription of antimalarials [15]. The population-level health benefits of incorporating community-based RDTs into malaria programmes is unclear, with some studies finding no difference in malaria-associated hospitalizations and mortality[15] and still others finding improvements in mortality among children under the age of five in SSA [34]. Still, effective RDT delivery may has the opportunity to improve malaria diagnosis and care, should they be well-integrated with existing community- and facility-based health services. Moreover, programmes seeking to expand access to self-testing via RDTs must consider how these pair with established malaria surveillance systems, including whether additional laboratory-based validiation is warranted, and how self-testing results may be reported for quantifying national and sub-national malarial burdens. Therefore, future investigations must evaluate delivery modalities for RDT-facilitated self-testing in ways that promote connectivity to the health system, rather than further complicate access by exacerbating barriers to care. These investigations must also be paired with engagement and planning with policymakers to account for the cost of procuring population-level coverage of RDTs, establishing reliable supply chains to ensure test availability, and guarantee timely regulatory approval of RDTs for self-testing.

This study has several limitations and strengths. First, as a cross-sectional study, this analysis may be subject to seasonal bias across these three SSA countries, limiting the generalizability of this study’s findings. Second, few children were enrolled in Zambia and South Africa. This is likely due to the day-time enrollment of study participants, during which time children may have been in school and unavailable for participation. This limits the study’s ability to draw conclusions about RDT self-testing for this vulnerable group, as it is challenging to determine the degree to which the sampled children are representative of the larger pediatric population at risk of malaria. Therefore, broader efforts to assess feasibility RDT self-testing for children in these populations are recommended. Third, the different sites employed different recruitment strategies. Particularly in Kenya and Zambia where sites were strategically, randomly selected, it is possible that the recruited households were not perfectly representative of the broader community. These selected households may have been more likely to benefit from RDTs or have better connectivity to CHW-led programmes, which may affect their reported acceptability to RDTs, although it is challenging to be certain about this dynamic. In contrast, in South Africa, randomly selected households may better represent the lower underlying prevalence of malaria, but these same households may also be less likely to see the value in RDTs for self-testing. Future studies may consider purposively sampling participants based on disease risk and access to care when determining patient-defined values such as acceptability. Fourth, as previously described, participants were not able to operate the RDT themselves due to regulatory approvals, but rather observed the RDT being used by trained study team members. As a result, it is possible that user preferences and acceptability are overestimated; future studies must allow participants to use RDTs themselves before evaluating user experiences. Among the study’s strengths is the large sample size across multiple SSA settings, providing deeper insight into community members’ preferences and perceptions. An additional strength is this study’s multifaceted approach to evaluating usability and acceptability of RDTs for new audiences.

## Conclusion

In conclusion, RDTs for self-testing were highly usable and acceptable; therefore, self-testing for malaria using RDTs is feasible in three sub-Saharan African settings. Additional research is recommended to understand effective methods to link people with positive RDTs to clinical care and treatment.

## Data Availability

The datasets used and/or analysed during the current study are available from the corresponding author on reasonable request.
